# Digital Leadership: Attributes of Modern Healthcare Leaders

**DOI:** 10.7759/cureus.21969

**Published:** 2022-02-07

**Authors:** Abdullah T Alanazi

**Affiliations:** 1 Health Informatics, King Abdullah International Medical Research Center, Riyadh, SAU; 2 Health Informatics and Public Health, King Saud Bin Abdulaziz University for Health Sciences, Riyadh, SAU

**Keywords:** innovation, strategic management, clinical information system, leadership, it system, digital technology

## Abstract

Background

Living in the current digital era requires widespread adoption of information technology in the modern healthcare industry.

Objective

The current research aimed to study key attributes and behaviors related to successful leaders' need to achieve the vision and ensure successful health IT adoption.

Methods

A Delphi technique with three rounds was employed and guided by structured questions. Part of the study was conducted online due to COVID-19 guidelines on distancing norms and lockdown in some areas. The answers of the participants were evaluated on a five-point Likert scale.

Results

The findings showed that similar leadership qualities are required in the healthcare sector as well as other sectors. For digital innovations in the rapidly changing healthcare space, leaders need to play a more proactive role, be visionary and dynamic, and should lead by example to take the organization to the next level.

Conclusions

Leaders need to come out of their comfort zone, understand the fast-evolving scenario where outstanding leadership qualities are essential to prove their mettle, outshine others, and create a strong foundation for the adoption of modern, efficient, customized digital technology in the fast-growing healthcare sector.

## Introduction

Successful leaders understand that the adoption of digital technologies can transform their organizations. El Sawy OA et al. de-emphasized the individualistic impact of the leader. They defined digital leadership as doing the right things for the strategic success of digitalization for the enterprise and its business ecosystem [1]. Currently, healthcare industry leaders are confronted with several challenges, such as financial constraints, maintaining high service quality, and a better standard of care. There is an urgent need for more operational efficiencies to maintain the utmost safety requirements for caregivers and patients and provide innovative information technology solutions in the modern healthcare system.

Westerman G et al., highlighting the role of a leader in executing the digital transformation, stated that a digital leader is an individual who mobilizes the organization through generating proper digital awareness and who possesses powers of influence over the people [2].

Information technology was introduced on a large scale in the 1950s. Health information technology (HIT) implies any clinical information system that is employed to deliver care or support the real-time delivery of health-related information. Adoption of HIT involves development, implementation, consolidation, continuous use, and iterative improvement in the system to ensure uninterrupted service delivery. It results in the adoption of several standards of care and overall improved outcomes in spheres such as organizational (e.g., measures of performance) or clinical (e.g., patient outcomes) fields. However, HIT projects have mostly failed because around three-fourths of all the digital projects exceed the allotted budget, take more time, deliver with less promised features, and have cost overruns as a norm rather than an exception. Several reasons contribute to the failure of HIT projects. These reasons include faulty planning process causing lack of clarity in terms of project's scope, failure to identify and engage relevant stakeholders in different stages of the project, lack of effective communication within the project team and with relevant stakeholders external to the team, and inability to identify and mitigate the risk effectively [3,4,5]. All these factors, especially the inability to effectively outline the project's scope, would also result in a situation where the planned budget will not reflect the real cost; therefore, these projects would be anticipated to run over budget [6]. Organizational maturity to adopt innovation and implement HIT solutions is one critical factor that determines the successful implementation of HIT solutions [7]. There are several examples of effectively adopting a HIT solution or a change undertaken systematically. American Well is one such example where a clear vision resulted in formulating a product that would facilitate virtual clinical services to the patients [8].

Effective leadership is crucial for introducing required reforms in healthcare. It, therefore, becomes essential to ensure the requisite capacity building of the digital leaders. Unfortunately, due to different factors, many leaders have failed to keep pace with the fast-evolving demands and timely delivery of services and outcomes in HIT implementation [9-10]. Because of the dynamic environment and recent advances in healthcare technology, the role of healthcare leaders has widened [9-11]. Traditionally, healthcare leaders were expected to ensure competency in clinical healthcare services and proper management. However, now, the leaders need to possess adequate knowledge of digital technologies related to health information and become change agents and innovators.

Avgar AC et al. studied the association between behaviors of leaders at the strategic and operational levels and IT adoption. As a result, a framework for understanding IT adoption in healthcare was postulated [12]. Ingebrigtsen T et al. found that communication of clear visions and goals of the organization by the leaders to all stakeholders and affected people and addressing work process change are associated with successful outcomes [13].

Several studies have observed that clear communication of vision positively impacted system adoption [14-15]. For example, in a scoping review, Laukka E et al. identified the significant role of healthcare leaders as supporters, change managers, and advocates for HIT implementation [16]. Similarly, a case study pertaining to outlining a systematic process to purchase a HIT solution shows how systems can be visionary and ambitious to ensure innovation but ensure due diligence and caution to avoid causing more harm than good, considering the complex nature of healthcare organizations [17].

Sheridan PT et al. analyzed the practice of leadership in health information management through the application of Bowen's theory. They outlined the main traits required of leaders: to be knowledgeable; have a better skill to manage finances; and understand project management timeline, resources, and alternative options [18]. Heath ML and Porter TH evaluated the traits of a physician leader to help them bridge the huge gap in expectation and delivery of outcomes and create collaboration in a digitized environment [19]. These traits include the development of trust among physicians, promoting their involvement, and engendering value proposition and a spirit of healthy competition [19].

Casimir G and Waldman DA suggested considering leadership attributes in the right context. At the same time, Lituchy TR et al. have observed notable differences among several groups in terms of traits possessed by effective leaders [20-21]. Carmen Leong LY and Fischer R documented significant differences in leadership attributes across different cultures and highlighted the contextuality of the leadership attributes [22].

The concepts of 'vision' and 'influence' that underpin the digital leader can be found in transformational leadership theory that discusses the endeavors of a leader to influence their followers in a stable environment. However, Jakubik M and Berazhny I, in their research, placed little emphasis on the method to lead in these data-driven, fast-paced, and ambiguous times. Therefore, exploration of the recommended leadership styles and traits of innovative leaders in the HIT sector is worth investigating and can add tremendous value to the body of knowledge for a better future of healthcare leaders [23].

## Materials and methods

One Delphi survey was conducted in three rounds. It included expert panelists, questionnaires used to collect data, and different tools employed for consensus-building. The survey aimed to generate innovative ideas from expert panelists by utilizing their relevant knowledge. This was done in order to gain first-hand experience in health services management. It enabled the participants to communicate their opinions and share knowledge anonymously and frankly. It also gave them the opportunity to review their opinions, understand the alignment of their ideas with others, and change their opinions, if necessary, after reformulating their ideas consistent with those of the group members. The expert panelists held leadership positions in the different healthcare sectors (the Ministry of Health and other tertiary hospitals, including government and private ones). A database of senior leadership positions was created to enlist the expert panelists. Purposive sampling was used to include knowledgeable and experienced participants in HIT projects. Twenty-three expert panelists were enlisted, and candidates' participation was also sought in the survey. Out of 23 panelists, 17 (74%) participated in the second round of the survey, and all 23 (100%) panelists participated in the third round. The survey was conducted between February 2020 and September 2020.

Round-one questionnaire development

Suitable data collection tools were developed. The first questionnaire had the following two sections: Section 1 contained demographic data about the panelists concerning the number of years of experience in the current position and the presence of other traits relevant to achieving the objective of the current study. Section 2 contained a questionnaire aimed at eliciting innovative ideas from the panelists on the leadership attributes necessary for digital leaders relevant, especially for completion of HIT projects within given budgets, deadlines, and set guidelines. In addition, open-ended questions were also asked to elicit more digital information by asking what leadership attributes are essential in today's leaders to tackle HIT projects?

Round two and three of the questionnaire surveys

The second and third rounds of the survey aimed to evaluate and build consensus among the participants on the leadership attributes identified from previous rounds to retain takeaways for the final round. Due to the COVID-19 lockdown and distancing norms, virtual meetings took place. The participants were advised to evaluate the concepts presented to them in the context of their inputs in the first round and modify their views considering the opinions of others and to agree or disagree with these concepts. The concepts had the following categories: · Turnaround leaders · Strategic manager · Effective communicator and possessing other outstanding interpersonal skills. The second questionnaire contained closed-ended questions and employed the five-point Likert scale to collect data related to their degree of agreement or disagreement (strongly agree = 5; agree; undecided; disagree; strongly disagree = 0) with the concepts presented. The battery of questionnaires was consolidated by combining similar responses and retaining the most important ideas. The five-point Likert scale was used to evaluate the panelists and indicate their degree of agreement or disagreement with the concepts presented.

Data collection process

All expert panelists were contacted by mail or phone and were informed about the study methodology. The first questionnaire was dispatched via email. Two email reminders were sent. No reminders were sent after six weeks from the first mail. The methodology employed in the second and third rounds involved online meetings due to the COVID-19 pandemic. The participating panelists in the first round partook in the second round and participants who responded to the second round were recruited for the third round.

Data analysis

The first questionnaire sought the generation of ideas from the panelists and had unstructured but qualitative data. These data were transcribed and thematically analyzed to identify recurring themes submitted by the participants. The documents were examined for related similar or different themes. One of the researchers undertook independent analysis in the dataset collection phase, and an assistant was made responsible for ensuring inter-rater reliability. The themes identified were employed to develop items for the next round of questionnaires. The second and third rounds sought to develop consensus on leadership traits required for successful HIT. The data in all rounds contained panelists' evaluation and reevaluation of their ideas in line with group summaries and descriptive statistics.

## Results

Demographic data were collected during the first round. Most of the panelists had more than 10 years of rich experience in the healthcare sector in managerial positions from three main cities across Saudi Arabia, namely Riyadh; 10 leaders (two females and eight males), Western province; four leaders (one female and three males) and Eastern province; three leaders (all males). The number of IT projects that participated in the study ranged between three and 18. The participants comprised three Chief Executive Officers (CEO), two Chief Medical Directors, five Chief Information Officers (CIO), three Information Directors, and four IT leaders with project management experiences.

Table 1 below exhibits the required attributes for digital leadership. The second and third rounds focused on building consensus on the identified attributes. As a result, a profound degree, i.e., greater than 92% of consensus, was evident in all the leadership attributes noticed in the first round of the study.

**Table 1:** Required attributes for digital leadership (n = 23).

Leadership attributes			Round 2, n = 17			Round 3, n = 23		
			Agreement (%)	Mean	SD	Agreement (%)	Mean	SD
Turnaround Leaders		Have transformational leadership attributes	100	1.62	0.34	100	1.64	0.28
		Proactive and take initiative	100	1.58	0.42	100	1.62	0.32
		Outcome-driven	100	1.52	0.32	100	1.58	0.36
		Accept responsibility	100	1,48	0.21	100	1.64	0.38
		Innovative	100	1.62	0.26	100	1.62	0.28
Strategic management	Analytical	Attention to detail	94	1.44	0.38	96	1.46	0.32
		Critical thinking	100	1.44	0.32	100	1.44	0.38
		Defining mechanisms for input	92	1.32	0.26	94	1.38	0.28
		Defining the purpose of the strategic planning process	100	1.64	0.36	100	1.62	0.34
		Developing a plan for implementing strategies.	94	1.62	0.32	94	1.62	0.32
	Decisiveness	Delegating	92	1.58	0.34	96	1.58	0.28
		Building Consensus	100	1.46	0.32	96	1.48	0.34
		Establishing Measurable Objectives for Goals/Projects	94	1.58	0.28	94	1.54	0.32
		Creating and Enforcing Timelines	96	1.44	0.26	96	1.44	0.28
		Prioritizing	92	1.42	0.32	96	1.46	0.34
		Goal-Oriented	100	1.62	0.28	100	1.54	0.36
		Confidence	90	1.44	0.34	96	1.48	0.38
Communication and interpersonal skills		Clearly articulate issues	100	1.66	0.24	100	1.62	0.28
		Effective communicators: listening, speaking, writing	100	1.68	0.20	100	1.64	0.26
		Effective interpersonal skills	98	1.68	0.22	98	1.62	0.28
		Effective in collaborating and cooperating	96	1.62	0.24	98	1.62	0.34
		Be team players	94	1.52	0.26	96	1.54	0.38
		People skills	100	1.62	0.32	100	1.58	0.36

## Discussion

Leaders in the digital arena should envision an ambitious and bold future for their respective organizations. They need to be innovative within the bounds of possibility and link these innovations to healthcare to improve the quality of care or cost reduction goals. The current study shows that certain leadership attributes are essential to enable a leader to participate in HIT innovation effectively. These attributes are turnaround leaders, strategic managers, effective communicators, and other interpersonal skills. A significant degree of consensus among the participants on the key leadership attributes in this regard (84-100%) was observed in the current study. Day G and Moorman C defined the turnaround strategy of a leader as the changes that are rapidly carried out as the company needs to respond to issues such as performance decline or downward trend in market share [24]. Intervention and support from the top management are prerequisites as the turnaround process requires resources, and it impacts the long-term sustainability of any organization. There is no one great leadership factor in turnarounds because every leader is different. However, good qualities for turnaround leaders include attributes such as the ability to influence, being visionary, inspiring a shared vision, being proactive, ability to take initiative to formulate strategies, ability to accept responsibility, ability to avoid blaming others and being innovative. These leaders know that just because a style or strategy was successful in the past does not mean it would be successful in the current situation as well.

Good strategic management is defined as formulating a strategy with clear-cut objectives, making specific plans about how the management objectives may be achieved, how the healthcare activities may be aligned to support the objectives, and how the resources need efficient allocation within permitted budgetary constraints to achieve the objectives. In a digital leader in modern healthcare systems, strategic planning skills encompass analytical ability, foresight, and decisiveness.

Analytical skills require that people working in strategic planning can evaluate the healthcare organization's plan. A leader needs to capitalize on his or her skill sets, step ahead after considering all factors to succeed, and be information savvy in a digitized world where clients' expectations are consistently rising. A leader needs to synergize, consolidate, improvise, and reach corporate objectives with better HIT systems to keep pace with these rising expectations. Leaders require attributes such as full attention to detail, critical thinking, defining mechanisms for input, defining the objective of the strategic planning process, developing a plan for implementing strategies, and being focused. It is especially important to understand the complexity of healthcare and anticipate the possible challenges this complex system can present while formulating any HIT solution. 

Strategic planning involves working decisively yet flexibly to meet the challenge at hand. Following skill sets are essential in good visionary leaders: delegation; building consensus; establishing measurable objectives for projects; creating and enforcing timelines; prioritizing; goal-oriented; confidence-building; and management by objectives.

Effective leaders articulate the vision and inspire people to follow a visionary path. For this, the healthcare leaders should ensure that the vision of the HIT project aligns with the organizational mission, purpose, and values. Interpersonal skills are the competencies necessary to interact effectively with other people. Essential interpersonal skills are conflict resolution, flexibility, empathy, teamwork.

Leading by example and learning from others

Recent literature on leadership shows three main approaches to being an effective leader: traits, behavioral and situational approaches. Bennis WG (1998) defines leaders as "people who can express themselves fully. They know what they want, why they want it, and how to communicate what they want to others, to gain their co-operation and support and they know how to achieve their goals".

One such example is a project led by Chaudhry R, where natural language processing was used to formulate a decision support system for screening and surveillance of cervical cancer [25]. In this case, the research team effectively communicated the need for such a system, highlighting that only 15% of the clinicians correctly recommended the screening tests. Therefore, identification of this problem clarified the scope of the problem. Additionally, effective communication within the research team resulted in coming up with an innovative way to conceptually design and evaluate the formulation of a surveillance system that is based on a novel technology like natural language processing. The development of such an innovative clinical decision support system shows effective communication between data scientists, clinicians, and statisticians. Though healthcare is complex, these innovations also require that the complexity of the clinical processes be effectively documented based on evidence and clinical guidelines so that innovative intervention is effectively designed and implemented. Furthermore, such intervention also requires that the architecture of the new clinical decision support system is effectively delineated and communicated as is done in the report of this project [25]. Finally, this clinical decision support is built on the routinely collected data; therefore, this project has effectively shown how to ensure interoperability between existing systems and the new decision support system [25].

The second example is the case study that discussed the processes adopted by Stanford Medicine while deciding whether to buy the new electronic health record (EHR) from Flatiron or not [17]. In this case, though, the decision seemed simple because of Flatiron's superior ability to provide analytical support to the organization and low cost. However, while making this decision, Stanford's IT leadership had the challenge to be strategically correct. It was important to choose an EHR system that can be adopted across different medical institutions at Stanford while ensuring continuous improvement of patient outcomes. To streamline this decision, Stanford's IT leadership outlined a systematic approach to make this decision to ensure that all these aspects are taken into consideration before any decision is made.

The next example is American Well, founded by Ido Schoenberg and his brother [8]. This was a platform that was one of the first in the world to bring patients and doctors together virtually for a clinical consultation. This platform was made after formulating a clear vision, communicating the vision to the team, and making a realistic plan after studying the healthcare environment, especially the structure of the healthcare delivery system in the United States.

Healthcare leaders need to design changes during the implementation of a turnaround strategy. This should include the information system, culture, and employees' attitudes [26]. Every employee in the organization should properly understand these changes. This builds trust toward the leader and the turnaround result [27]. Lawson E and Price C further highlight the need to precisely define the transformation process and effectively communicate every element of the hospital [28].

Limitations

The generalizability of the results of this study should be approached cautiously as the study was conducted in a limited setting. Nevertheless, the participants came from the three main cities of Saudi Arabia and represent the majority of HIT leadership in the country. Furthermore, the author has utilized the purposive sampling technique to assure that potential leaders are approached.

Additionally, the participants were predominantly males, possibly due to the male dominance in the higher echelons of Saudi Arabia. Besides, the Delphi approach fails to define the consensus clearly. However, this study's consensus level of agreement was remarkably high, at 92% (in the second round) and 94% (in the third round). This might have resulted in several key issues getting ignored. Besides, the regrouping of the individual attributes into three categories (turnaround leaders, strategic management, and communication and interpersonal skills) needs further assessment. It should be tested for its consistency through confirmatory factor analysis in future studies.

## Conclusions

Different digital leadership styles have remained unexplored and understudied by researchers and scholars so far. The current study showed that the leadership style required to transform healthcare digitally resembled facets of existing leadership theories. The study aimed to develop consensus on the required leadership traits for participation in health policy development. Overall, it can be concluded that turnaround leaders, strategic managers, communicators, and those with excellent interpersonal skills can prove digital leadership in the HIT sector. This study also found that successful leaders understand that digital technologies may transform their entire healthcare and can articulate a vision about preferred transformation for successful digital leadership.
